# Effects of Ketogenic Diet on Neuroinflammation in Neurodegenerative Diseases

**DOI:** 10.14336/AD.2021.1217

**Published:** 2022-07-11

**Authors:** Ziying Jiang, Xi Yin, Miao Wang, Tong Chen, Yuanyuan Wang, Zhongbao Gao, Zhenfu Wang

**Affiliations:** Department of Neurology, The Second Medical Center and National Clinical Research Center for Geriatric Disease, Chinese PLA General Hospital, Beijing 100853, China

**Keywords:** ketogenic diet, ketone bodies, neuroinflammation, neurodegenerative diseases, experimental and clinical evidence

## Abstract

The ketogenic diet (KD) is a low-carbohydrate, high-fat and adequate-protein diet. As a diet mimicking fasting, it triggers the production of ketone bodies (KBs) and brings the body into a state of ketosis. Recent and accumulating studies on humans and animal models have shown that KD is beneficial to neurodegenerative diseases through modulating central and peripheral metabolism, mitochondrial function, inflammation, oxidative stress, autophagy, and the gut microbiome. Complicated interplay of metabolism, gut microbiome, and other mechanisms can regulate neuroinflammation in neurodegenerative diseases by activating multiple molecular and cellular pathways. In this review, we detail the physiological basis of the KD, its functions in regulating neuroinflammation, and its protective role in normal brain aging and neurodegenerative diseases, such as Alzheimer’s disease (AD), Parkinson’s disease (PD), amyotrophic lateral sclerosis (ALS), and Huntington’s disease (HD). We aimed to elucidate the underlying neuroinflammatory mechanisms of KD therapies in neurodegenerative diseases and provide novel insights into their application for neurodegenerative disease prevention and treatment.

The ketogenic diet (KD) is a high-fat, low-carbohydrate, and moderate protein dietary intervention [1]. As one of the forms of fasting, KD can make the organism substitute fat for carbohydrates as an energy source. During the KD, fatty acids are converted to ketone bodies (KBs) by liver metabolism and then enter the bloodstream to induce nutritional ketosis and participate in subsequent physiological or pathological reactions [2,3]. KD was initially established as an alternative therapy for refractory epilepsy in the 1920s [4]. Adapted versions of the KD are currently widely applied to treat obesity [5], diabetes [6], and age-associated diseases [7]. Considering the safety and accessibility of KD, exogenous ketone supplements (KS) have been developed to mimic the physiological effects of KD on various diseases [8,9].

Neurodegenerative diseases, such as Alzheimer's disease (AD), Parkinson's disease (PD), amyotrophic lateral sclerosis (ALS), and Huntington's disease (HD), are characterized by progressive and chronic loss of the structure and functions of neuronal materials [10,11]. The etiologies and pathogenesis of neurodegenerative diseases are complicated and varied, and include protein aggregations, genetic mutations, and infections. The discovery of chronic activation of the immune response in the central nervous system (CNS) and elevated levels of inflammatory factors in patients and animal models suggest that neuroinflammation plays a remarkable role in the complex pathogenesis of neurodegenerative diseases [12]. The key pathological hallmarks of neuro-inflammation are activation and proliferation of the major CNS immune cells, microglia and astrocytes, which are accompanied by the regulation and release of inflammatory mediators [13,14]. As there is currently neither a cure nor an effective disease-modifying therapy for most neurodegenerative diseases, developing effective strategies for therapies is crucial. Recently, emerging evidence has underlined both pathophysiological and clinical benefits of KD in neurodegenerative diseases, indicating that KD is a possible treatment option for neurological illnesses [15]. KD can exert protective effects by modulating multiple neuroinflammatory pathways [16,17]. In this review article, we aimed to detail the physiological basis of KD and to summarize the current data regarding the possible neuroinflammatory mechanisms of KD in various neurodegenerative disorders. We also provide an overview of the current experimental and clinical evidence supporting the therapeutic potential of KD for normal brain aging and neurodegenerative disorders specifically targeting neuroinflammation.

## Biochemistry and physiological function of KD

### Brief history of KD

**Figure 1.**
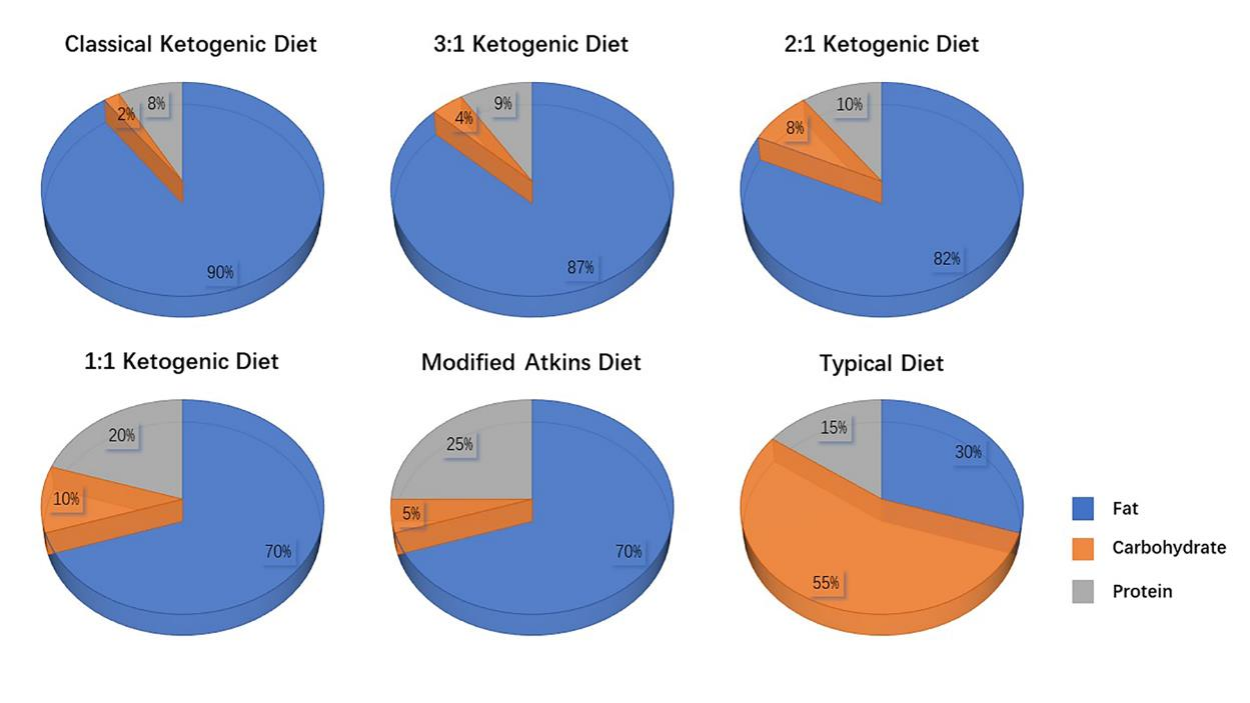
Characteristics of the classical ketogenic diet and its common modifications.

In 1921, Woodyatt et al. found that the levels of KBs, beta-hydroxybutyric acid (βHB), and acetone were elevated in healthy subjects who followed fasting or a low carbohydrate and high-fat diet. Concurrently, Wilder et al. confirmed that a special high-fat and low carbohydrate-based diet contributed to improved seizure control in drug-resistant epilepsy patients. These significant findings might reveal the origin of the classical KD [18]. The prominent features of the classical KD are a high fat content and a low carbohydrate content, with a macronutrient ratio of fat to protein and carbohydrate standing at 4:1. The total daily carbohydrate intake is limited to 20-50 g, which can provide 5%-10% of the total energy intake per day. The carbohydrate restriction inhibits the increase in insulin levels, which in turn promotes the conversion of fatty acids to KBs (βHB, acetoacetate, and acetone) and provides approximately 90% of the dietary energy in the classical KD [1]. The classical KD has been modified to enhance its palatability and flexibility, such as the modified Atkins diet (MAD) [19] and lower ratio KD (3:1, 2:1, and 1:1) [20].Figure 1 shows the macronutrient composition of the classical KD and its common modifications. The ultimate goal of the KD is the production of KBs, and subjects can test the levels of KBs through blood or urine samples to ensure the adequacy of their dietary intervention [21].

### Ketogenesis in the liver

Under normal conditions, humans obtain energy through glycolysis, the conversion of glucose to pyruvate. However, under conditions of nutrient deprivation, such as dietary carbohydrate restriction, fasting, KD, and prolonged physical exercise, the flexible human metabolism triggers ketogenesis and produces KBs as a substitute for glucose (the main metabolic fuel). This metabolic process occurs primarily in the hepatic mitochondrial matrix [22] as well as in extra-hepatic mitochondria (astrocytes and kidneys), albeit to a lesser degree [23]. This metabolic process is depicted inFigure 2. Increasing glucagon and epinephrine levels and decreasing insulin levels induce lipolysis from the adipose tissue and release fatty acids into the bloodstream by enhancing the key enzyme activities in these processes (hormone-sensitive lipase, adipose lipase enzymes, and adipose triglyceride lipase). The free fatty acids (FFAs) are enzymatically converted into fatty acyl-coenzyme A (acyl-CoA) and further transported into hepatocellular mitochondria through the carnitine palmitoyl transferase (CPT) [24,25]. The increasing acetyl-CoA level via β-oxidation in the hepatic mitochondrial matrix is far beyond the metabolic ability of the Krebs cycle, also named the tricarboxylic acid (TCA) cycle, which subsequently initiates ketogenesis [26]. With a chain of biochemical reactions, acetyl-CoA is broken down into acetoacetate by thiolase, 3-hydroxymethylglutaryl-CoA (HMG-CoA) synthase 2 (HMGCS2), and HMG-CoA lyase (HMGCL) [27]. Acetoacetate is reduced to βHB by β-hydroxybutyrate dehydrogenase 1 (BHD1), and finally, both βHB and acetoacetate are delivered to the bloodstream or target organs through monocarboxylate transporters (MCTs) [28]. Additionally, acetone is produced from a small part of acetoacetate through spontaneous decarboxylation and then metabolized in the liver. Under normal physiological conditions, both βHB and acetoacetate are excreted by the kidneys, whereas acetone is excreted by the lungs [29,30].

Due to the lack of critical enzymes for ketolysis in the liver, βHB and acetoacetate are released into the circulatory system to be metabolized in non-hepatic oxidative tissues, including the brain, skeletal muscle, heart, and kidney. Under healthy conditions, the plasma βHB level is quite low (less than 0.3 mmol/L), whereas the glucose level is high (approximately 4 mmol/L) [7]. Under a state of carbohydrate limitation, a human liver can deliver 150-300 g of KBs into the circulatory system per day [2]. The ratio of βHB and acetoacetate levels in plasma is approximately 5:1, which can fluctuate according to the mitochondrial redox ratio in the liver [31,32]. After a 3-4 day fast or starvation, the βHB level in plasma rises to 5-6 mmol/L, which can meet ≥ 50% of the brain energy requirements [33]. After dietary intervention or diabetic ketosis, the βHB level may surge to > 25 mmol/L [34].

**Figure 2.**
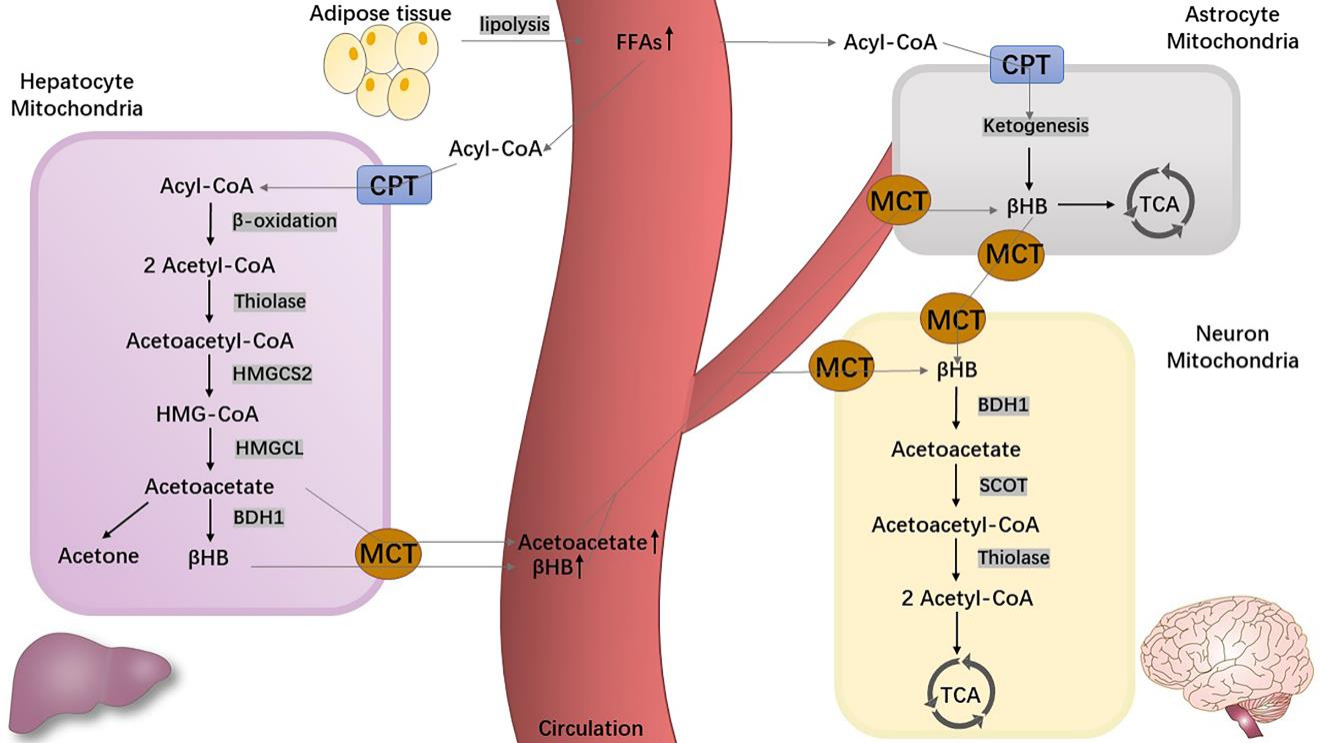
**The metabolism of ketone bodies**. Abbreviations: acetoacetyl-CoA, acetoacetyl-coenzyme A; acetyl-CoA, acetyl-coenzyme A; acyl-CoA, acyl-coenzyme A; βHB, beta-hydroxybutyric acid; BHD1, β-hydroxybutyrate dehydrogenase 1; CPT, carnitine palmitoyl transferase; FFAs, free fatty acids; HMG-CoA, 3-hydroxymethylglutaryl-CoA; HMGCL, 3-hydroxymethylglutaryl-CoA lyase; HMGCS2, 3-hydroxymethylglutaryl-CoA synthase 2; MCTs, monocarboxylate transporters; SCOT, succinyl-CoA-3- ketoacid CoA transferase; TCA, tricarboxylic acid.

### Ketolysis in the brain

At the start of dietary carbohydrate or energy restriction, the glucose stored in the brain can supply adenosine triphosphate (ATP) for only a few minutes [35]. Once the KBs achieve concentrations > 4 mmol/L, a switch from glucose to KBs occur in the brain energy source [36]. KBs can be taken up into the brain tissue via MCTs on micro-vascular endothelial cells and astrocytes, and the transport process depends on the concentrations of KBs in the circulation [15,37]. MCTs are a family of five proton-linked transporters that passively transport metabolic substrates, such as lactate, pyruvate, and KBs. The family contains four isoforms in mammals, including MCT1 (SLC16A1), MCT2 (SLC16A7), MCT3 (SLC16A8), and MCT4 (SLC16A3), each of which has characteristic substrates and affinities [28]. As the exclusive known transporters of KBs, MCTs are ubiquitously expressed in the brain. MCT expression in the brain is significantly upregulated following the administration of KD in rats [38,39]. MCT1 possesses a relatively low affinity for βHB, principally located in endotheliocytes and astrocytes of the blood-brain barrier (BBB) [40]. MCT2 has a high affinity for βHB and is almost exclusively located in the post synaptic density of neurons [41]. Additionally, low-affinity MCT2 is mainly located in astrocytes [42]. Interestingly, a recent study found that FFAs can cross the BBB and participate in ketogenesis in astrocytes, mediated by ventromedial hypothalamic (VMH) neurons [43]. Further, the endogenous KBs produced from astrocytes transfer to nearby neurons for energy supply, which the process is similar to the astrocyte neuron lactate shuttle (ANLS) [23]. Generally, neurons might be capable of βHB uptake, while glia cells might be capable of release or uptake of βHB.

The reverse reactions of ketogenesis, known as ketolysis, occur in the mitochondrial matrix. First, βHB is oxidized into acetoacetate by BHD1 following its arrival in mitochondria. Then, acetoacetate is catalyzed and catabolized to two molecules of acetyl-CoA to participate in the TCA cycle by the action of succinyl-CoA-3- ketoacid CoA transferase (SCOT) and thiolase [44]. Of note, the metabolism of KBs does not require ATP but can generate more energy than glucose [45,46]. Furthermore, studies on rodents have reported that the rate at which neurons, astrocytes, and oligodendrocytes can metabolize KBs to supply energy is higher than the rate at which they metabolize glucose. Compared to astrocytes, neurons and oligodendrocytes might achieve higher efficiency of KB oxidative metabolism [15,47]. The synthesis and catabolism of KBs involved in the brain are illustrated inFigure 2.

### Neuroprotective effects of KD

KD plays neuroprotective roles in the CNS through inhibition of glycolysis and the production of KBs. First, as an essential feature of dietary restriction, inhibition of glycolysis regulates insulin secretion and improves insulin sensitivity and glucose tolerance, which consequently delays the onset of age-related conditions and prolongs the lifespan of various species [17,48]. Second, KBs compensate for mitochondrial dysfunction in neurons and glial cells by improving mitochondrial respiration and increasing mitochondrial ATP production [49]. A previous study suggested that βHB catabolism regulates nicotinamide adenine dinucleotide (decreasing the NAD+/NADH ratio), while coenzyme Q (increasing the Q/QH2 ratio) couples the balance in βHB-perfusion of rat hearts. The vast difference in the redox potentials of these two couples facilitates the electron transfer process from NADH to Q and ATP production during oxidative phosphorylation [50]. Furthermore, the interaction between α-synuclein aggregation and mitochondrial function may involve blockade of complex I (CI) in the pathomechanism of PD [51]. Production of succinate, an oxidative substrate of complex II (CII), is regarded as the rate limiting step of βHB catabolism. Studies have shown that βHB metabolism can circumvent pathological CI inhibition by increasing flux through CII by generating succinate in dopaminergic neurons [52,53]. Third, a KD alleviates the production of reactive oxygen species (ROS) and abates inflammatory responses through various signaling pathways. Fourth, a KD may be able to prevent neuronal apoptosis via the sirtuin (SIRT)-1 signaling pathway. A KD reduces the SIRT-1-mediated hyperacetylation of the transcription factor p53 and consequently down-regulates the proapoptotic protein (BAX) and up-regulates the anti-apoptotic proteins (Bcl-2 and Bcl-xL) in the Cockayne syndrome animal model [54].

### Strategies for nutritional ketosis induction

Ketosis is a physiopathologic process that is achieved by disturbances in the carbohydrate metabolism, owing to KD or dietary carbohydrate restrictions. In contrast to diabetic ketoacidosis, nutritional ketosis is a safe and sustained physiological state with plasma βHB concentrations ranging from 0.5 to 5 mmol/L [55,56]. The physiological characteristics of nutritional ketosis are normal blood gas, elevated KBs, and low and stable glucose. Besides KD, fasting, medium-chain fatty acids (MCFAs), and exogenous ketones can also induce nutritional ketosis. With the development of medical science, prolonged fasting has been modified into alternating and intermittent variations of fasting to achieve nutritional ketosis safely [57]. Another alternative to KD is the intake of exogenous MCFAs, which are absorbed rapidly into the portal vein and preferentially enter β-oxidation and ketogenesis in the liver [58]. MCFAs are composed of caprylic acid (C8) and capric acid (C10), which are found chiefly in coconut and palm kernel oil [6]. Dietary supplements of exogenous ketones, such as ketone salts and esters in the form of dissolvable powders or liquids, also induce ketosis [59]. Exogenous ketones directly increase the circulatory pool of KBs and accurately manipulate the KB levels with no decline in insulin or glucose levels [60,61].

### Limitations of KD

Despite the widely recognized protective role of a KD, its suitability and safety need to be evaluated in further research. A major limitation of the application of the KD is the use of KBs in targeted organs, especially in the brain. The kinetic profile of KBs appears to be strongly affected by the formulation and dose of KD therapies. A clinical study showed that the maximum plasma levels of βHB and acetoacetate were achieved within 15 min in healthy subjects after the injection of DL-β-hydro-xybutyrate or acetoacetate. The fractional rate constant of disappearance (K), volume of distribution (V), and metabolic clearance (MCR) of of βHB and acetoacetate are 66 and 52 × 10^-3^ min^-1^, 28.3 and 30.6 L, and 1.59 and 1.58 L/min, respectively [62]. In another similar study, 54 healthy subjects received (R)-3-hydroxybutyl (R)-3-hydroxybutyrate orally to evaluate the kinetic parameters of this burgeoning βHB monoester [8]. The βHB monoester at 357 and 714 mg/kg allowed maximum plasma levels of KBs to be achieved safely in 1-2 h, which was similar to those observed following fasting for several days. It is recognized that the use of KBs in an organism is determined by the concentration of KBs in the bloodstream. Although there is a linear relationship between the usage rate and blood concentration at lower concentrations of KBs, there is a plateau when the usage rate reaches the maximum at higher concentrations of KBs [63]. KBs, especially βHB, are readily transported through the BBB via MCTs to participate in brain energy metabolism [64]. Brain uptake of βHB and acetoacetate increases frequently during KD therapies or short-term starvation [65], which seems to be related to the alternation of BBB permeability [66], transporters [65], and capillary density [67]. However, the KB concentrations in the brain and cerebrospinal fluid were much lower than those in the bloodstream during starvation or KD therapies [63]. Thus, a new ketogenic therapy that is more easily absorbed and used by the brain deserves further research and development.

An additional concern of the applicability of the KD is patient compliance with particular dietary recommendations [7]. Although patients are initially determined and motivated to comply with the KD, they are prone to abandon dietary treatment due to poor tolerance to a large number of fat-rich foods in the later stage. Therefore, we should consider whether intermittent KD therapy may offer beneficial effects analogous to those observed with the standard KD schedule. This speculation has been recently evaluated, and the results confirmed that intermittent KD weekly prevents obesity and improves midlife survival and memory in aging mice [68].

As multiple methods of nutritional ketosis induction have been increasingly used for neurologic disorders, concern and research into potential adverse effects have gradually increased. A clinical study of patients with epilepsy treated with KD reports the adverse effects including dyslipidemia, gastrointestinal effects (nausea, vomiting, constipation and diarrhea) and weight loss [20]. Moreover, KD may lead to dehydration, hypoglycemia, hyponatremia, atherosclerosis, impaired hepatic functions, and deficiencies of multiple nutrient elements [69]. Of note, anorexia and acute pancreatitis are considered a contraindication of KD therapies. Approaches are recommended to prevent and control the side effects of KD, such as having small, frequent meals (SFMs) throughout the day, increasing intake of dietary fiber, vitamins, minerals, and water, and performing more exercise [20,70]. Taken together, a standardized treatment protocol for KD that details the dose and duration of dietary intervention, accurately monitors the KB level, and prevents potential adverse effects remains to be established.

## Possible anti-inflammatory mechanisms of KD

Inflammation is a comprehensive array of biological processes that occur in response to foreign organisms and injury suffered by cells or tissues. A rapid and effective inflammatory response eliminates invading pathogens, promotes wound healing and angiogenesis, and ultimately repairs damaged tissues. Neuroinflammation is defined as an inflammatory process that occurs in the brain or spinal cord and is caused by diverse pathological lesions, such as trauma, infection, ischemia, and degeneration [71]. Resident CNS glial cells (microglia and astrocytes) and peripheral infiltrating immune and endothelial cells mediate neuroinflammation by regulating the production of inflammatory cytokines, ROS, chemokines, and small-molecule messengers. According to the course of the inflammatory response, neuroinflammation can be divided into acute and chronic. Acute neuroinflammation, an immediate response to damaging stimuli, induces beneficial effects resulting from the removal of possible destructive factors, phagocytosis of cell debris, and maintenance of homeostasis. However, uncontrolled neuroinflammation induces a chronic inflammatory response, which is characterized by microglia overactivation, production of neurotoxic factors, and destruction of neurons and glial cells, which together accelerate the deterioration of the condition [72]. The context, course, and duration of the main stimulus or injury affect the degree of these neuroinflammatory responses to a large extent.

Microglia and astrocytes are the innate immune cells primarily involved in neuroinflammation [73,74]. Microglia are the resident macrophages in the brain and are key players in neuroinflammation [75]. Microglia have pro-inflammatory or neuroprotective roles in the CNS depending on their phenotype (proinflammatory M1 phenotype and anti-inflammatory M2 phenotype) and activation pathways. The M1-type microglia are activated via nuclear factor-κB (NF-κB) and signal transducers and activators of transcription 3 (STAT3) signaling pathways to generate pro-inflammatory cytokines, such as interleukin (IL)-1β, IL-6, IL-12, IL-23, cyclooxygenase (COX)-1, COX-2, ROS, and nitric oxide (NO). In contrast, the M2-type microglia are activated to release neurotrophic factors, such as IL-4, IL-10, IL-13, and transforming growth factor-β (TGF-β). Astrocytes are the most common type of glial cells and can regulate cerebral blood flow, maintain neurotransmitter homeostasis, control synapse formation, and form distinctive perivascular channels to eliminate potentially neurotoxic agents. Similar to microglia, activated astrocytes have dual phenotypes (A1 phenotype and A2 phenotype) to respond to pathological insults and participate in the neuroinflammatory process. The proinflammatory A1-type astrocytes are induced by inflammatory insult and produce pro-inflammatory cytokines (such as IL-1β, TNF-α, IL-6, and NO) via the NF-κB signaling pathway. The anti-inflammatory A2-type astrocytes are induced to release anti-inflammatory cytokines (such as IL-4, IL-10, IL-13, and TGF-β) via the STAT3 pathway.

It has been implicated that the neuroinflammatory process contributes to the pathology of several neurodegenerative diseases [76]. As resident neuroimmune cells of the CNS, microglia and astrocytes modulate the inflammatory response and release pro-inflammatory or anti-inflammatory cytokines in response to abnormal accumulation of pathogenic proteins (such as tau-containing neurofibrillary tangles in AD and α-synuclein aggregation in PD) and invasion of pathogens [77]. It is accepted that both central (such as pathological protein deposition and brain injury) and systemic (such as infections, dietary patterns, and lifestyles) effector specificity mechanisms can result in CNS neuroinflammation. There is mounting evidence to suggest that a KD plays neuro-protective and disease-modifying roles in neurodegenerative diseases by regulating both central and peripheral inflammatory mechanisms [20]. We expound the multi-dimensional potential mechanisms involved in the anti-inflammatory properties of a KD inFigure 3.

**Figure 3.**
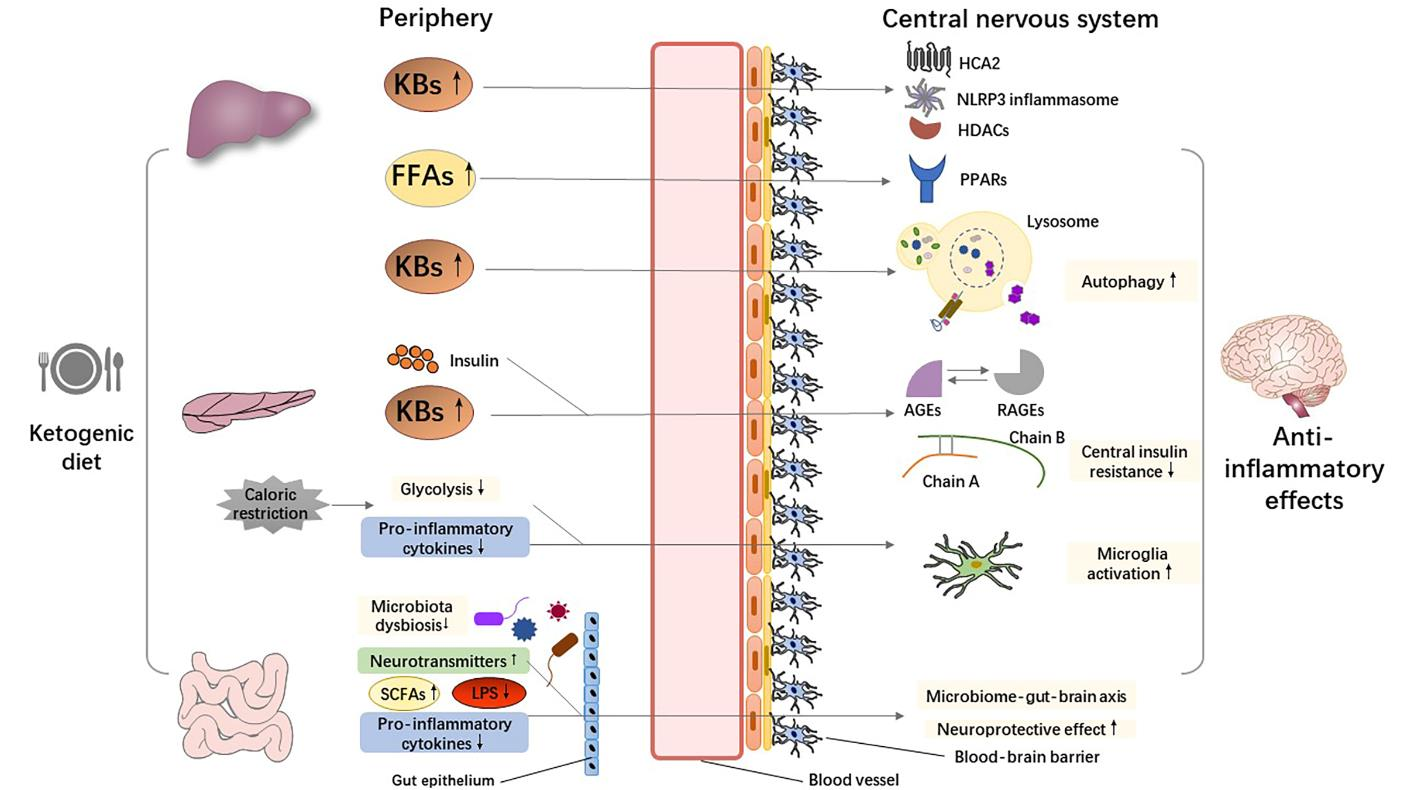
**Putative anti-inflammatory mechanisms mediated by the ketogenic diet**. Abbreviations: AGEs, advanced glycation end products; FFAs, free fatty acids; HCA2, hydrocarboxylic acid receptor 2; HDACs, histone deacetylases; KBs, ketone bodies; LPS, lipopolysaccharide; NLRP3, nucleotide-binding domain-like receptor protein 3; PPARs, peroxisome proliferator-activated receptors; RAGEs, receptors for AGEs; SCFA, short chain fatty acid.

**Figure 4.**
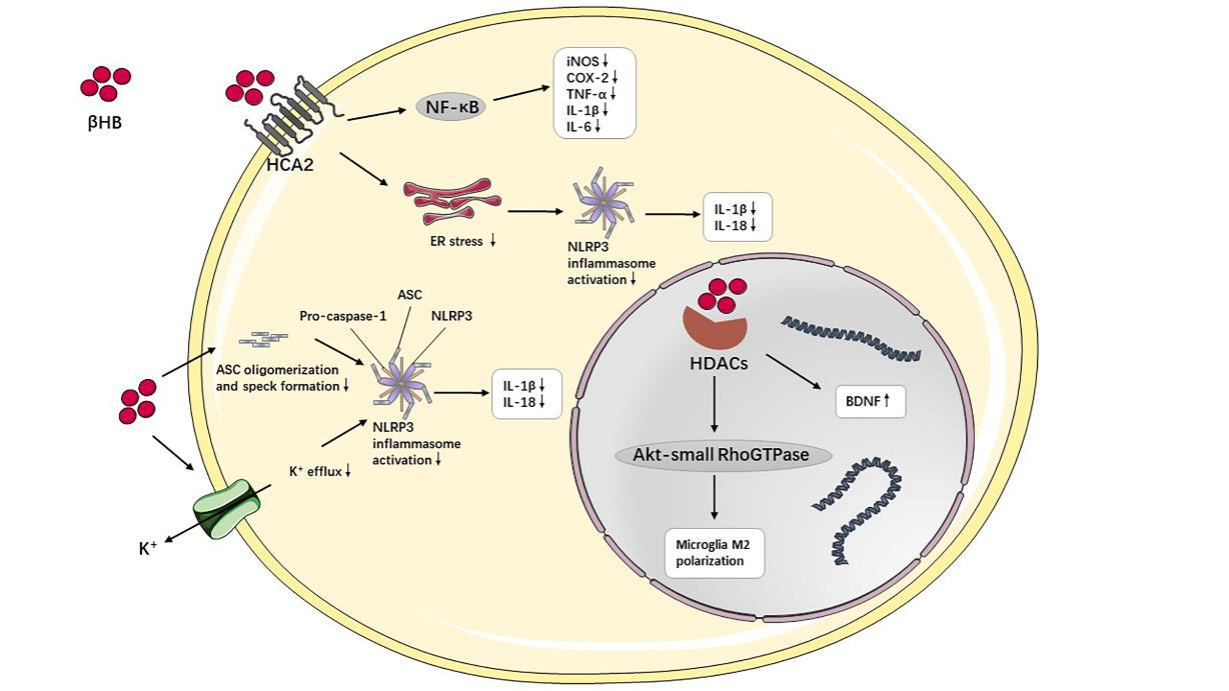
**The intracellular molecular mechanism of neuroinflammation mediated by beta-hydroxybutyric acid (βHB)**. βHB interacts with hydrocarboxylic acid receptor 2 (HCA2), nucleotide-binding domain-like receptor protein 3 (NLRP3) inflammasome and histone deacetylases (HDACs) directly or indirectly to exert anti-inflammatory effects. Abbreviations: ASC, apoptosis-associated speck-like protein with a caspase recruitment domain; BDNF, brain-derived neurotrophic factor; βHB, beta-hydroxybutyric acid; COX, cyclooxygenase; ER, endoplasmic reticulum; HCA2, hydrocarboxylic acid receptor 2; HDACs, histone deacetylases; IL, interleukin; iNOS, inducible nitric oxide synthase; NF-κB, nuclear factor-κB; NLRP3, nucleotide-binding domain-like receptor protein 3; TNF-α, tumor necrosis factor-α.

### KBs as signal mediators

Besides being energy substrates, KBs are also active as intracellular signaling mediators, which participate in intracellular signaling cascades and regulate neuroinflammation directly or indirectly, especially βHB [78].Figure 4 shows the putative anti-inflammatory mechanism of βHB mediated by cell-signaling pathways. Hydrocarboxylic acid receptor 2 (HCA2), also named G-Coupled Protein Receptor (GPR) 109A, is a G protein-coupled receptor that is expressed abundantly in microglia, macrophages, and dendritic cells. The activation of HCA2 triggers the neuroprotective subset of macrophages dependent on COX-1 generating prostaglandin D2 (PGD2), which serve to relieve neuroinflammation [70]. As a specific endogenous ligand of HCA2, βHB is capable of directly binding to HCA2 and activating it with a half maximal effective concentration (EC50) of approximately 0.7 mmol/L, which is similar to that in human nutritional ketosis [79,80]. βHB can bind to HCA2 to further inhibit the production of pro-inflammatory cytokines and enzymes via the NF-κB pathway in activated primary microglia pretreated with βHB and stimulated with lipopolysaccharide (LPS) [81]. However, the activation of HCA2 by βHB also eases the inflammatory response through partly reducing endoplasmic reticulum (ER) stress, nucleotide-binding domain-like receptor protein 3 (NLRP3) inflammasome activity, and IL-1β and IL-18 levels [82].

Additionally, the innate immune sensor, the NLRP3 inflammasome, is widely expressed in the cytoplasm of immune cells to modulate neuroinflammation and can be activated by the alternation of cytoplasmic K^+^ levels. βHB inhibits the activation of the NLRP3 inflammasome and subsequently IL-1β and IL-18 generation by blocking K^+^ efflux and decreasing oligomerization and speck formation of apoptosis-associated speck-like protein with caspase-recruitment domain (ASC). Of note, the interaction between βHB and the NLRP3 inflammasome is independent of the oxidation in the TCA cycle, HCA2, SIRT-2, and uncoupling protein-2 (UCP-2) [83].

Data suggest that βHB can engage with histone deacetylases (HDACs) to alter the expression of detoxifying genes at the epigenetic level. βHB inhibits class I HDACs (1, 2, 3, and 8) and increases the expression of brain-derived neurotrophic factor (BDNF) [45,84]. It is well recognized that BDNF induces the activation of microglia and astrocytes, thereby contributing to neuroinflammation through various signaling pathways [85]. The inhibition of HDAC activity by βHB may have other downstream actions. For example, βHB triggers the M2 microglia phenotype, which is reversed by HDAC suppression, potentially via the Akt (protein kinase B)-small RhoGTPase (Rac1, Cdc42) axis [86].

### Peroxisome proliferator-activated receptors (PPARs) activation

PPARs, including PPARα, PPARβ, and PPARγ, are nuclear receptors that act as ligand-inducible transcription factors to modulate lipid and glucose metabolism. PPARα expression is constitutively found in the liver, heart, and kidney, while PPARβ is mainly expressed in the brain, skin, and adipose tissue. PPARγ is also expressed in the adipose tissue, brain, muscle, and various tissues [87,88]. PPARs are highly upregulated in the brains of study animals under a ketotic state, which may result from the activation of PPARγ co-activator 1α (PGC-1α) and SIRT-1 or KBs [89,90]. Moreover, PPARα and PPARγ play a leading role in the regulation of ketogenesis and the ketolysis process, both of which can be activated by KD-induced elevated polyunsaturated fatty acids in the periphery and brain [68,91,92]. Previous studies have suggested that MCFAs, as a component of certain KDs, are direct agonists of PPARγ [93,94]. Although the mechanisms need to be explored in more depth, current evidence suggests that a KD can play an anti-inflammatory role by activating PPARs. Indeed, PPARα activated by FFAs has been shown to inhibit the NF-κB signal transduction pathway, leading to downregulation of inducible nitric oxide synthase (iNOS) and COX-2 to modulate the inflammatory response [95]. There is some evidence to confirm that the activation of PPARγ induced by a KD has a marked suppressive effect on neuroinflammation in the hippocampus of a mouse model of seizures [96]. Moreover, the activation of PPARs reduces the inflammation response in the periphery and brain, which seems to result from the restraint of NF-κB, nuclear factor of activated T-cells (NFAT), and signal transduction and transcription-1 (STAT-1) [97,98]. The elevated PPARγ level blocks STAT-1 and p38 mitogen-activated protein kinase (MAPK) signaling and impedes the appearance of a reparative or pro-inflammatory microglia phenotype, thereby facilitating the transition of microglia to anti-inflammatory phenotype [99,100]. Interestingly, the activation of PPARγ seems to exert more prominent effects in facilitating the anti-inflammatory microglia phenotype under conditions of increased FFA levels, which might be involved in the mammalian target of rapamycin (mTOR)-PPARγ signaling pathway [101].

### Autophagy regulation

Autophagy is a tightly orchestrated cellular process mediated by lysosomes, which degrades and eliminates intracellular endogenous components (misfolded or aggregated proteins and damaged organelles) and exogenous stimuli (bacteria, virus, and parasites). Autophagy has been subdivided into three major types according to their distinct patterns of cargo delivery to the lysosome: macroautophagy, microautophagy, and chaperone-mediated autophagy (CMA) [102,103]. A growing body of evidence suggests that autophagy is closely linked to aging and to the onset and progression of neurodegenerative diseases [104-106]. Recent studies have demonstrated that autophagy limits inflammatory pathologies and alleviates the burden of infectious agents to exert anti-inflammatory and neuroprotective effects [107,108].

The KD, the βHB component to be exact, might ease inflammatory reactions mediated by autophagy in neurodegenerative diseases. During glucose deprivation, elevated βHB stimulates autophagy and improves cortical neuronal survival [109]. Nutritional ketosis can up-regulate macroautophagy in the brain through inhibition of the mechanistic target of rapamycin complex 1 (mTORC1) and activation of sirtuin-1 (Sirt1) and hypoxia-inducible factor-1 (HIF-1) [110,111]. βHB promotes CMA activation directly by increasing protein oxidation, which ultimately leads to the clearance of oxidation-damaged proteins [112]. Additionally, βHB attenuates autophagic degradation and improves neuronal damage induced by N-methyl-D-aspartate (NMDA) in the striatum of rat [113]. Compared to the cerebral cortex, more autophagosome formation occurs in the hippocampus in response to a KD [114]. Interestingly, HMGCS2, the control enzyme in ketogenesis, induces autophagic clearance of the β-amyloid precursor protein (APP), which is mediated by acetoacetate metabolism and the mTOR signaling pathway; this suggests that HMGCS2 inhibits the subsequent production and deposition of amyloid β (Aβ) [115].

### Insulin sensitivity improvement

Insulin resistance and hyperglycemia are associated with brain aging and neurodegenerative diseases and can aggravate the pathological process through a complex network of feedback mechanisms, especially in AD and PD [116]. Insulin resistance may be induced by diabetes, hypertriglyceridemia, obesity, and systemic chronic inflammation [117]. Moreover, the reduction of glucose transporters and disturbance of insulin signaling pathways contribute to insulin resistance at the molecular level [118]. Insulin resistance and impaired glucose metabolism accelerate the formation of advanced glycation end products (AGEs), which can bind to receptors for AGEs (RAGEs) on neurons and glia cells to exert harmful effects. The activation of RAGEs facilitates the expression of pro-inflammatory cytokines, such as IL-1, IL-6, and tumor necrosis factor-α (TNF-α), along with ROS production, which further results in neuroinflammation, oxidative stress, and neuronal degeneration [119,120]. Recent experimental and clinical studies have demonstrated that a KD can improve insulin sensitivity as well as glucose homeostasis [121-123]. Further studies have shown that βHB, the main KBs in the brain, restrains insulin glycation, AGE accumulation [124,125], and liposomal lipid peroxidation to ultimately prevent microglial apoptosis [126]. Thus, we can speculate that the anti-inflammatory effects of KD could hinder the development of neurodegenerative diseases through increasing insulin sensitivity.

### Caloric restriction

The implementation of KD has shown some weight loss effects by reducing food intake and increasing energy expenditure [127]. Although controversial, KD can be linked to caloric restriction in some cases [7]. As a basic characteristic of calorie restriction, suppression of glycolysis might alter microglial phenotypes and exert anti-inflammatory action by regulating the NAD+/NADH ratio and the transcriptional inhibitor C-terminal-binding protein (CtBP) [92,128]. Caloric restriction can also modulate the expression of inflammatory regulators, such as NF-κB inhibitor, PPARs, and tissue inhibitor of metalloproteinases-3 (Timp3) [129,130], and down-regulate the levels of TNF-α, IL-2, IL-4, IL-6, COX-2, and iNOS [131]. Additionally, the anti-inflammatory effects of calorie restriction are likely to be caused by various epigenetic regulations, including microRNA [132]. The upregulation of miR-16-5p, miR-196b-5p, and miR-218-5p has been reported in the serum of calorie-restricted mice, and notably, miR-16-5p significantly reduced TNF-α, IL-1β, and IL-6 levels after LPS stimulation [133]. Some reviews have elaborated on the possible mechanisms of the calorie restriction anti-inflammatory properties of KD [17,134].

### Gut microbiota modulation

Considering that gut microbes have an influence on central behavior and pathology, the concept of the microbiome-gut-brain axis has been proposed recently. The gut microbiota involves nervous, immune, and endocrine mechanisms and is termed the “second brain” [135,136]. The gut microbiota influences the host CNS via various neurotransmitters (such as norepinephrine, dopamine, and γ-aminobutyric acid) in the gut. Dietary features, both total calorie and individual nutrients (such as carbohydrate, fat, protein, and vitamins), are considered to have a major impact on microbiota composition. While diet-induced alterations occur over time, diet-induced alterations of the gut microbiota may happen rapidly if shifting diets are sufficiently dramatic [137]. It has recently been suggested that KD-induced gut microbiota profile changes also mediate neuro-inflammation [16].

There is accumulating evidence to support that KD alters the gut bacterial profile in both animal models and human studies. Indeed, after 16 weeks of KD treatment, the neurovascular functions, including BBB function and cerebral blood flow, were shown to be sufficiently improved to accelerate Aβ clearance in young healthy mice. This KD intervention also altered the composition of the gut microbiota, with enrichment of beneficial microbes (such as Lactobacillus and Akkermansia muciniphila) and a reduction of pro-inflammatory microbes (such as Turicibacter and Desulfovibrio) [138]. A previous clinical study demonstrated that patients with mild cognitive impairment (MCI) have a relatively high abundance of Enterobacteriaceae and Erysipelotriaceae but a relatively low abundance of Lachnobacterium and Bifidobacterium after a modified Mediterranean-ketogenic diet (MMKD). The MMKD also reduces acetate and lactate in feces and increases butyrate, which is a beneficial short-chain fatty acid (SCFA), to exert neuroprotective actions [139]. In previous in vivo and in vitro experiments, compared to the high-fat diet, KD resulted in the suppression of bifidobacterial growth through the host producing βHB. The gut microbiota induced by KD down-regulate pro-inflammatory Th17 cells and, consequently, the Th17-associated immune response in the intestine, which might explain the potential protective mechanism of KD in metabolic syndrome and neurological diseases [140]. The abnormally altered gut microbiota, known as gut microbiome dysbiosis, is associated with neuro-degenerative diseases. Gut microbiome dysbiosis decreases the levels of neurotransmitters and SCFAs and increases the level of LPS, ultimately adjusting the interplay between the CNS and gut [141]. Moreover, the gut microbiome dysbiosis contributes to intestinal inflammation and intestinal barrier dysfunction, further leading to elevated levels of inflammatory cytokines and LPS in circulation [142]. KD promotes the production of SCFAs and restrains the production of γ-glutamyl amino acid through altering particular microbes, such as Lactobacillus and Akkermansia muciniphila [136,141]. Considering the lack of studies of KD on gut microbiota, more research is needed to confirm the anti-inflammatory role of KD in the CNS.

## Effects of KD on normal brain aging and neurodegenerative diseases

The majority of previous studies have examined the effects of KD on normal brain aging and neurodegenerative diseases. KD is increasingly regarded as an alternative therapy for a range of neurodegenerative diseases. In this section, we summarize the main published experimental and clinical evidence related to the role of KD in normal brain aging and neurodegenerative diseases (presented inTable 1), especially the involvement in neuroinflammatory mechanisms.

**Table 1:** Preclinical and clinical studies on ketogenic diet in normal brain aging and neurodegenerative diseases.

Models	Intervention	KD effects on inflammatory or other brain pathology markers	KD effects on clinical features	Study 1st author (ref)
**Normal brain aging**				
**Animal models**	KD; medium chain triglyceride	Modulates the synaptic stability and synaptic plasticity	Improves cognitive function	Newman JC [68]; Pan Y [144]; Wang D [146]
	KD	Alters expression transporters for different energy substrates and neurotransmitters	Enhances motor performance	Hernandez AR [145]
**AD**				
**Animal models**	KD; ketone ester	Reduces Aβ and hyperphosphorylated tau deposition	Relieves anxiety, improves cognitive function	Van der Auwera I [153]; Kashiwaya Y [154]
	βHB; triheptanoin	Inhibits NLRP3 inflammasome activation, microgliosis and reduces plaque formation; inhibits astrogliosis and pro-inflammatory cytokines production, improve mitochondrial status; reduces APP and increases NEP mediated by GPR109A	Improves cognitive function	Shippy DC [155]; Aso E [156]; Wu Y [157]
	Ketone ester; 3-Hydroxybutyrate methyl ester	Promotes TCA cycle metabolites and decreases mitochondrial redox potential; reduces Aβ deposition, protects mitochondrial functionality and corrects the intracellular redox state, inhibits cell apoptosis	Improves the spatial learning and working memory;	Pawlosky RJ [158]; Zhang J [159]
	βHB	Inhibits cell apoptosis with the reduction of p53, caspase-3, caspase-9, caspase-12 levels and the Bax/Bcl-2 ratio		Xie G [160]
	KD, ketone ester	Elevates the level of n-acetyl-aspartate	Improves the motor performance, does not improve cognitive performance; improves the abnormal behaviour	Brownlow ML [161]; Pawlosky RJ [162]
**Human**				
	Medium chain triglyceride; MMKD	Increases cerebrospinal fluid Aβ42 and decreases tau protein mediated by the alteration of gut mycobiome, gut bacteria and SCFAs, increases cerebral perfusion and cerebral KBs uptake	Improves memory in subjects with MCI	Rebello CJ [164]; Neth BJ [163]; Nagpal R [165]; Nagpal R [139]
	KD; medium chain triglyceride; MAD		Improves the quality of life and daily function; Ameliorates cognitive impairment, especially in the APOE ɛ4 negative patients	Phillips MCL [166]; Ota M [167]; Brandt J [168]; Henderson ST [169]; Reger MA [170]; Ohnuma T [171]
	Caprylic triglyceride		Dose not improve functional ability or cognitive impairment	Henderson ST [172]
	Medium chain triglyceride	Enhances brain ketone uptake and energy supply; enhances rCBF in specific brain regions		Croteau E [173]; Torosyan N [174]
**PD**				
**Animal models**	βHB; C8	Attenuates the loss of dopamine, improves mitochondrial respiration and ATP production, up-regulates the expression of PGC-1α and PEPCK	Improves the motor deficits	Tieu K [52]; Joniec-Maciejak I [182]
	KD	Increases Nissl and tyrosine hydroxylase-positive neurons, increase dopamine and dihydroxyphenylacetic acid and glutathione		Cheng B [183]
	KD; βHB	Protects dopaminergic neurons, inhibits GPR109A, NF-κB signal pathway, microglial activation and downstream pro-inflammatory cytokines production	Improves the motor dysfunction	Yang X [184]; Fu SP [81]
	KD	Dose not protect dopaminergic neurons		Kuter KZ [185]
**Human**	KD		Improve the motor and non-motor symptoms, especially cognitive function; improve the voice quality	Vanitallie TB [186]; Phillips MCL [188]; Koyuncu H [187]
**ALS**				
**Animal models**	KD	Protects motor neurons, promotes ATP production and mitochondrial respiration	Enhances the motor performance	Zhao Z [191]
	Triheptanoin; Caprylic triglyceride	Increases mitochondrial oxygen consumption	Improves the motor functions, delays the onset of motor symptoms and increases the survival	Tefera TW [192]; Zhao W [193]
	Deanna protocol		Enhances the motor performance and increases the survival	Ari C [194]
**HD**				
**Animal models**	KD		Attenuates motor deficits and slow down the loss of weight	Chen JY [196]
	βHB	Attenuates striatal lesions and microgliosis, inhibits histone deacetylation	Ameliorates motor dysfunction, extends the lifespans	Lim Soyeon [197]

Abbreviations: Aβ, amyloid β; AD, Alzheimer’s disease; ALS, amyotrophic lateral sclerosis; APOE ɛ4, epsilon 4 allele of the apolipoprotein E gene; APP, β-amyloid precursor protein; ATP, adenosine triphosphate; βHB, beta-hydroxybutyric acid; C8, caprylic acid; GPR, G-Coupled Protein Receptor; HD, Huntington’s disease; KBs, ketone bodys; KD, ketogenic diet; MAD, modified Atkins diet; MCI, mild cognitive impairment; MMKD, modified Mediterranean-ketogenic diet; NEP, neprilysin; NF-κB, nuclear factor-κB; NLRP3, nucleotide-binding domain-like receptor protein 3; PD, Parkinson’s disease; PEPCK, phosphoenolpyruvate carboxylase; PGC-1α, peroxisome proliferator-activated receptor γ co-activator-1α; rCBF, regional cerebral blood flow; SCFA, short chain fatty acid; TCA, tricarboxylic acid.

### KD and normal brain aging

Brain aging is relevant to cognitive decline and is considered a dominant risk factor for neurodegenerative diseases, including AD, PD, and vascular dementia [143]. Several studies have explored the effects of KD on cognitive function and synaptic plasticity in aged animals [68,144-146]. Indeed, some KD methods, including traditional KD and exogenous KS, have demonstrated a positive effect on cognitive function in aged animals. Moreover, medium chain triglyceride diets have been shown to increase Ube3a protein expression and down-regulate arc, *plk3, junb, egr2*, and *nr4a1*genes to modulate synaptic stability and synaptic plasticity in aged rats [146]. Additionally, KD was found to contribute to the alteration of transport protein expression, including glucose, monocarboxylates, vesicular glutamate, and γ-aminobutyric acid transporters, in the hippocampus of aged rats, which might correct age-related abnormal energy metabolism, synaptic transmission, and neurotransmitter systems [145].

### KD and AD

AD is the most common neurodegenerative disease and the most common cause of dementia in elderly people and is manifested by memory decline and subsequent mental and behavioral impairment, with insidious onset and slow progression [147]. The core histopathological hallmarks of AD are extracellular deposition of Aβ plaques, intracellular hyperphosphorylation, and abnormal aggregation of tau protein. There are more than 25 million people with dementia worldwide, most of whom have AD. The prevalence of dementia has notably increased, leading to onerous burdens on individual patients, caregivers, and society in both developed and developing countries [148]. MCI is regarded as a transitional stage between normal aging and dementia; therefore, it is crucial to find effective prevention therapies to delay the onset of MCI and slow cognitive decline [149].

Several studies have confirmed that βHB inhibits Aβ-induced neurotoxicity [150] mediated by facilitating Aβ efflux across the BBB [151], restraining HDAC1/3, and up-regulating the level of tyrosine kinase receptor A (TrkA) [152]. Several studies have shown that various types of KD can lessen Aβ, hyperphosphorylate tau deposition, and improve cognitive function in transgenic mouse models of AD [153,154]. First, βHB improved cognitive performance and attenuated related pathology by targeting diverse inflammatory mechanisms in AD models. βHB inhibited NLRP3 inflammasome activation, microgliosis, astrogliosis, and pro-inflammatory cytokine production [155-157]. βHB modulated β-amyloid precursor protein (APP) and neprilysin (NEP) expression mediated by GPR109A to suppress the amyloid plaque formation in 5xFAD (familial AD) mice [157]. Second, ketone ester significantly contributed to supplying energy by KB metabolism, protecting mitochondrial functionality, and correcting the intracellular redox state in transgenic AD mice [156,158,159]. Third, βHB directly inhibited the apoptotic signaling pathway with the reduction of p53, caspase-3, caspase-9, caspase-12 levels, and the Bax/Bcl-2 ratio [159,160]. Tg4510 (a model of tau deposition) and APP/PS1 (a model of amyloid deposition) mice fed a KD for 3 months performed significantly better on the motor tests, compared to those fed a normal diet, although this was not the case in cognitive tests [161]. A KD was also shown to play a protective role in the improvement of abnormal behavior, including avoidance-related behavior and exploratory activity, by elevating the level of n-acetyl-aspartate in the hippocampus of 3xTgAd mice [162].

Several studies have found relationships between the KD and cognitive decline in humans. Indeed, medium chain triglyceride supplementation for 24 weeks or the MMKD for 6 weeks appeared to improve memory in subjects with MCI in two randomized controlled trials [163,164]. The MMKD could regulate the biomarker levels of AD with increasing Aβ42 and decreasing tau protein in cerebrospinal fluid, which might be mediated by the alteration of gut mycobiome, gut bacteria, and SCFAs [139,165]. Likewise, clinical trials suggested that different types of KDs significantly improved the quality of life and daily function [166] and eased AD-related cognitive impairment [167,168], especially in subjects without the epsilon 4 allele of the apolipoprotein E gene (APOE ɛ4), suggesting that KB metabolism is related to APOE ɛ4 status [169-171]. However, a multicenter clinical trial found that the AC-1204 formulation of caprylic triglyceride had no significant effect on improving functional ability or cognition among 413 patients with AD and without the APOE ɛ4 allele [172]. Some studies could explain the mechanisms underlying the protective effect of KD on AD. Medium chain triglyceride supplements triggered brain ketone uptake and energy supply to compensate for the brain energy deficit caused by hypometabolism among patients with AD [173]. However, caprylidene, a medium chain triglyceride, enhanced regional cerebral blood flow (rCBF) in specific brain regions, including the temporal cortex, cerebellum, and hypothalamus, among patients with AD and without the APOE ɛ4 allele [174].

### KD and PD

PD is the second most common progressive neurodegenerative disorder characterized by motor disturbances (such as resting tremors, bradykinesia, muscle rigidity, and slowness of movement) and non-motor disturbances (such as sleep dysfunction, autonomic dysfunction, hyposmia, cognitive, and psychiatric symptoms) [175]. The core neuropathological hallmarks of PD are substantial loss of dopaminergic neurons in the substantia nigra pars compacta (SNpc), along with the accumulation of α-synuclein, known as Lewy neurites or Lewy bodies [176]. As the fastest-growing neurological disorder, the number of people with PD is projected to increase to more than 12 million in the coming decades, driven primarily by the aging population [177].

Several experimental studies have shown that βHB has a neuroprotective effect in several cell models of PD through various mechanisms, including attenuation of mitochondrial dysfunction [150] via modulating CI or CII [178] and diminishing synphilin-1 aggregation [179], inhibition of apoptosis via increasing the ratio of Bcl-2/Bax mRNA [180], and regulation of neuroinflammation via inhibition of NLRP3 inflammasome activation [181]. βHB administered subcutaneously conferred protection partly against motor disturbance and reduction of striatal dopamine in mice induced by 1-methyl-4-phenol-1,2,5,6-tetrahydropyridine (MPTP), which might be related to a CII-dependent mechanism and easing of the mitochondrial respiration chain and ATP production [52]. Similarly, another study revealed that C8, one of the highest producers of KBs, significantly prevented dopaminergic neurodegeneration in the striatum of the MPTP mouse model of PD. Further study demonstrated that this appeared to be mediated by the up-regulation of PGC-1α and phosphoenolpyruvate carboxylase (PEPCK), which are regarded as markers of mitochondrial activity [182]. KD has also been shown to up-regulate glutathione of the striatum, thereby reversing the decrease in dopamine and dihydroxyphenylacetic acid in a 6-hydroxydopamine- (6-OHDA) induced rat model of PD [183]. KD was also shown to exhibit neuroprotective and anti-inflammatory effects via restraining microglial activation and reducing pro-inflammatory cytokines, such as TNF-α, IL-1β, and IL-6, in the MPTP mouse model [184]. Moreover, βHB interacted with GPR109A and suppressed NF-κB activation and downstream pro-inflammatory cytokine production, such as iNOS, COX-2, TNF-α, IL-1β, and IL-6, in LPS-treated in vivo and vitro PD models [81]. However, according to recent research, a long-term KD lasting for 7 weeks did not prevent 6-OHDA-induced damage to dopaminergic nigral neurons, indicating that the KD seemed to affect astrocytic energy support but not oxidative stress [185].

Few trials of KD in patients with PD have been implemented. A pilot study indicated that the Unified Parkinson’s Disease Rating Scale (UPDRS) scores were improved after five patients with PD adhered to a KD for 28 days [186]. Additionally, a 3-month KD increased the voice quality of patients, as evaluated by the voice handicap index of patients with PD [187]. In another pilot randomized controlled trial, 47 patients with PD were randomly assigned to a KD group and a low-fat group over 8 weeks, and the primary outcomes of motor and non-motor symptoms were assessed by the International Parkinson and Movement Disorder Society-sponsored UPDRS (MDS-UPDRS). The findings suggested that both diets could significantly improve the motor and non-motor symptoms, with the KD showing a greater effect on less L-dopa-responsive non-motor symptoms. Meanwhile, the randomized controlled trial confirmed that a low-fat diet or KD for patients with PD over 8 weeks was safe and reasonable, with excessive hunger being the most common adverse effect during the overall process [188]. Of note, a study discovered that KD might not disturb the pharmacokinetics of levodopa, as detected using the high-performance liquid chromatography method using UV detection (HPLC-UV) in patients with PD [189].

### KD and ALS

ALS is the most common motor neuron disease, characterized by the progressive loss of upper and lower motor neurons in the brain and spinal cord. The weakness or paralysis of the skeletal muscle results in motor dysfunction in both limbs and the bulbar region. Ultimately, death due to respiratory paralysis usually occurs within 2-5 years of the onset of the above-mentioned symptoms [190]. The first published research in 2006 showed that a KD significantly enhanced motor performance in SOD1-G93A transgenic ALS mice, which might be related to the preservation of motor neurons and promotion of ATP production and mitochondrial respiration [191]. Some triglycerides, including caprylic triglyceride and triheptanoin, could improve motor functions via the protection of motor neurons [192,193]. Specifically, caprylic triglyceride facilitated mitochondrial oxygen consumption [193], and triheptanoin delayed the related symptoms and improved the survival [192] in SOD1-G93A mice. Moreover, better motor performance and an increase in survival were observed in ALS mice under the Deanna protocol, a metabolic therapy derived from the KD [194].

### KD and HD

HD is a common autosomal dominantly inherited disorder caused by an abnormally expanded CAG trinucleotide repeat in the huntingtin gene on chromosome 4. HD is characterized by progressive uncontrollable dance-like movements and cognitive and psychiatric disturbance [195]. An investigation showed that symptomatic R6/2 mice raised on a KD lost weight more slowly, achieved higher scores in behavior tests, and had fewer stereotypes compared to R6/2 mice raised on a normal diet [196]. Additionally, when βHB was administered subcutaneously to the 3-nitropropionic acid (3-NP) toxic model of HD, the motor dysfunction, striatal lesions, and microgliosis were attenuated. Similarly, βHB extended the lifespan, alleviated motor dysfunction, and inhibited histone deacetylation of the striatum in R6/2 transgenic mice treated in the same way. Further research proved that βHB inhibited histone deacetylation through an HDAC-independent mechanism in PC12 cells with inducible expression of human mutant huntingtin [197]. Thus, additional investigations of KD effects in animal models and patients with HD are necessary.

## Conclusions and perspectives

The prevalence of neurodegenerative disorders is increasing owing to lifespan extension and unhealthy aging. Given that there is currently no cure nor a disease-modifying therapy available for neurodegenerative disorders, identifying preventive interventions and therapeutic strategies is crucial to delay the onset of neurodegenerative disease and slow progression. From the recognition that KD is not merely a traditional treatment of epilepsy but that is also plays a neuroprotective role in neurodegenerative disorders, it has been suggested that KD might be a promising way to protect against the multiple symptoms of neuro-degenerative disorders. Numerous studies have shown that KD contributes to energy metabolism, oxidative stress, neuroinflammation, and apoptosis in neurodegenerative diseases through pleiotropic mechanisms. In this review, we summarize the beneficial effect of KD in regulating neuroinflammation and provide a comprehensive overview of experimental and clinical evidence of KD in normal brain aging and neurodegenerative diseases.

Although the present findings suggest that KD offers a promising therapeutic approach for neurodegenerative disorders, further preclinical studies aimed at identifying the characteristic effects of KD on the pathophysiology of neurodegenerative disorders are needed. Additionally, further large-scale and long-term clinical research on neurodegenerative disorders, especially randomized control trials, is warranted to evaluate the efficacy, sustainability, and the adverse effects of KD. Choosing optimal approaches to inducing nutritional ketosis for individuals should be considered carefully in future clinical practice. Compared to the classical KD, exogenous KS or a lower ratio KD might be a relatively accessible way to achieve nutritional ketosis in many aspects. While there is still a long way to go, the prominent effects of KD suggest its use as a new therapeutic target and strategy for neurodegenerative disorders.
